# The potential of depressive symptoms to identify cognitive impairment in ageing

**DOI:** 10.1007/s10433-025-00837-1

**Published:** 2025-02-25

**Authors:** Panagiotis Alexopoulos, Christos Bountoulis, Everina Katirtzoglou, Mary H. Kosmidis, Kostas Siarkos, Mary Yannakoulia, Efthimios Dardiotis, Maria Skondra, Georgios Hadjigeorgiou, Robert Perneczky, Paraskevi Sakka, Eleni-Zacharoula Georgiou, Μarina Charalampopoulou, Panagiotis Felemegkas, Iracema Leroi, Apostolos Batsidis, Laura Perna, Antonios Politis, Nikolaos Scarmeas, Polychronis Economou

**Affiliations:** 1 Department of Medicine, School of Health Sciences, Mental Health Services, Patras University General Hospital, University of Patras, 26504 Rio, Patras, Greece; 2 Global Brain Health Institute, Medical School, Trinity College Dublin, Dublin, Republic of Ireland; 3 Department of Psychiatry and Psychotherapy, Klinikum Rechts der Isar, Faculty of Medicine, Technical University of Munich, Munich, Germany; 4 Patras Dementia Day Care Center, Patras, Greece; 5 Department of Business Administration, University of Piraeus, Piraeus, Greece; 6 1st Department of Psychiatry, Eginition Hospital, School of Medicine, National and Kapodistrian University of Athens, Athens, Greece; 7 Lab of Cognitive Neuroscience, School of Psychology, Aristotle University of Thessaloniki, Thessaloniki, Greece; 8 Department of Nutrition and Dietetics, Harokopio University, Athens, Greece; 9 Department of Neurology, Larissa University General Hospital, School of Medicine, University of Thessaly, Larissa, Greece; 10 Department of Neurology, Medical School, University of Cyprus, Nicosia, Cyprus; 11 Division of Mental Health in Older Adults and Alzheimer Therapy and Research Center, Department of Psychiatry and Psychotherapy, University Hospital, Ludwig-Maximilians-Universität Munich, Munich, Germany; 12 Ageing Epidemiology (AGE) Research Unit, School of Public Health, Faculty of Medicine, The Imperial College of Science, Technology and Medicine, London, UK; 13 German Center for Neurodegenerative Diseases (DZNE) Munich, Munich, Germany; 14 Munich Cluster for Systems Neurology (SyNergy), Munich, Germany; 15 Sheffield Institute for Translational Neurosciences (SITraN), University of Sheffield, Sheffield, UK; 16 Athens Association of Alzheimer’s Disease and Related Disorders, Maroussi, Greece; 17 Department of Mathematics, University of Ioannina, Ioannina, Greece; 18 Department of Genes and Environment, Max Planck Institute of Psychiatry, Munich, Germany; 19 Department of Psychiatry, Division of Geriatric Psychiatry and Neuropsychiatry, Johns Hopkins Medical School, Baltimore, USA; 20 1st Department of Neurology, Eginition Hospital, National and Kapodistrian University of Athens Medical School, Athens, Greece; 21 Department of Neurology, Taub Institute for Research in Alzheimer’s Disease and the Ageing Brain, The Gertrude H. Sergievsky Center, Columbia University, New York, NY USA; 22 Department of Civil Engineering, School of Engineering, University of Patras, Patras, Greece

**Keywords:** Geriatric depression scale, Adaptive boosting algorithm, Mild cognitive impairment, Dementia due Alzheimer’s disease

## Abstract

Depressive symptoms are common in mild cognitive impairment (MCI), dementia caused by Alzheimer’s disease (AD dementia) and in cognitively unimpaired older adults. However, it is unclear whether they could contribute to the identification of cognitive impairment in ageing. To assess the potential utility of depressive symptoms to distinguish between healthy cognitive ageing and MCI and AD dementia. The diagnostic workup of the cognitive function of 1737 older cognitively unimpaired individuals, 334 people with MCI and 142 individuals with AD dementia relied on a comprehensive neuropsychiatric assessment, including the Mini Mental State Examination (MMSE). Depressive symptoms were tapped with the 15-item Geriatric Depression Scale (GDS). Proportional odds logistic regression (POLR) models and the machine learning technique Adaptive Boosting algorithm (AdaBoost) were employed. Stratified repeated random subsampling (stratified bootstrap resampling) was used to recursive partitioning to training- and validation set (70/30 ratio). The average accuracy of the POLR models for the GDS total score in distinguishing between cognitive impairment and healthy cognitive ageing exceeded 78% and was inferior to that of MMSE. Of note, the sensitivity of GDS total score was very low. By employing the AdaBoost algorithm and considering GDS items separately, the average accuracy was higher than 0.72 and comparable to that of the MMSE, while sensitivity- and specificity values were more balanced. The findings of the study provide initial evidence that depressive symptoms may contribute to distinguishing between cognitive impairment and cognitively healthy ageing.

**Supplementary Information:**

The online version contains supplementary material available at 10.1007/s10433-025-00837-1.

## Background

Depressive symptoms are common across the entire spectrum of cognitive ageing. Worldwide the average prevalence in older individuals is about 32% (Zenebe et al. 2021). Among older people with cognitive deficits, which do not severely hamper performance on activities of daily living, (mild cognitive impairment; MCI), the prevalence of depressive symptoms is estimated to be 25% in community-based and 40% in clinic-based samples (Ismail et al. 2017). The prevalence rate of depressive symptoms in people with dementia is roughly 40% (Helvik et al. 2019; (Eliza) Georgiou et al. 2023). Interestingly, the most common alterations associated to late-life depression, such as neurotransmitter imbalance, hypothalamus–pituitary–adrenal (HPA) axis dysregulation, reduction of nerve growth factors, vascular disease and neuroinflammation are linked to pathological changes in the ageing brain, including Alzheimer’s disease (AD)-related pathology (amyloid accumulation, tau aggregation, neurodegeneration), and cerebrovascular pathologies, which often co-exist (Rahimi and Kovacs 2014; Alexopoulos 2019). Additionally, cortical amyloid load is higher in older people with depression and no dementia than in older individuals with neither dementia nor depression and the association between severity of depression and amyloid load follows a dose–response pattern (Smith et al. 2021). Despite the clinical and biological links between depression and cognitive decline in ageing, the potential contribution of depressive symptoms to the diagnostic workup of MCI and dementia caused by AD (AD dementia) has not been thoroughly studied yet. However, especially in light of forthcoming disease-modifying therapies for AD, it is of high relevance to explore whether depressive symptoms could contribute to the identification of individuals in the earliest stages of cognitive decline in order to screen people for biomarker testing and referral to specialist memory centres.

The aim of this study was to explore the potential utility of depressive symptoms in distinguishing between cognitive impairment and healthy cognitive ageing in a Greek cohort using machine learning methods. Interestingly, machine learning models, being a type of artificial intelligence, are advanced probabilistic and statistical techniques which can easily, and correctly identify data patterns and have recently emerged as useful tools in clinical practice for instance in prediction of chronic diseases and mental health crises (Garriga et al.; Delpino et al. 2022). We hypothesized that (i) depressive symptoms can contribute to the detection of cognitive impairment, with a utility comparable to that of the widely used screening test Mini Mental State Examination (MMSE) (Arevalo-Rodriguez et al. 2021); (ii) a machine learning technique, considering depression scale items separately together with demographics, amplifies the utility of depressive symptoms in differentiating between cognitive impairment and healthy cognitive ageing.

## Materials and methods

All study procedures were conducted in accordance with the ethical standards of the relevant national and institutional committees on human experimentation and with the Helsinki Declaration of 1975, as revised in 2008. They were approved by the Εthics and Research committees of the Eginition University Hospital (BEY846C8N2-32Π, 24/18.02.2013; ΩΖ5Ε46Ψ8ΝΖ-7Τ4, 04/24.01.2018), the General University Hospital of Larissa (138/08.07.2009) and of the General University Hospital of Patras (535/07.10.2021). All participants or authorized representatives gave their written informed consent.

### Participants

The study sample included 277 older adults who underwent a comprehensive diagnostic workup for cognitive decline at the old age psychiatry outpatient clinics either at the Eginition University Hospital in Attica or at the Patras University General Hospital in the Region of Western Greece, and 1,936 participants of the Hellenic Epidemiological Longitudinal Investigation of Ageing and Diet (HELIAD) (Alexopoulos et al. 2021a; Vlachos et al. 2021). HELIAD recruited older adults from urban, suburban and rural sites, who were randomly sampled and invited to participate in the study. Baseline data were collected between 2011 and 2016. The study sample is a convenience one. Its participants went through a standardized comprehensive neuropsychiatric assessment that is typical for tertiary healthcare settings. Inclusion criteria were (1) age 65 years or older, (2) diagnosis of MCI or AD dementia, or absence of a neurocognitive disorder, (3) available 15- item Geriatric Depression Scale (GDS)- and MMSE data (Fountoulakis et al. 2000, 2014; Kourtesis et al. 2020). Diagnoses were established based on international criteria for MCI and AD dementia (Petersen 2004; Albert et al. 2011; McKhann et al. 2011; Sachdev et al. 2014). Individuals in whom cognitive deficits or functional impairment was not detected, were classified as cognitively healthy. The diagnoses were established through consensus meetings of all clinicians/investigators involved in the diagnostic workup of each individual.

### Assessment of depressive symptoms and cognitive function

Depressive symptoms were captured with the 15-item GDS, a well-established, brief, self-report instrument for screening and evaluating depressive symptoms whose items require a yes/no response (Allgaier et al. 2011; Fountoulakis et al. 2014). Of note, the GDS does not include items related to the somatic symptoms of depression, which could be present in older individuals even in the absence of depression and subsequently embody a source of bias (Acosta Quiroz et al. 2021). The neurocognitive assessment included the MMSE (Fountoulakis et al. 2000; Creavin et al. 2016). It is a paper-and-pencil test, the administration of which does not exceed ten minutes. The MMSE has a maximum score of 30 points. It assesses orientation, learning, attention, memory, language and visual construction. According to a rule of thumb that is commonly employed in clinical practice, MMSE scores lower than 24 points indicate the presence of dementia, while scores ranging between 24 and 27 points are compatible with MCI (Fountoulakis et al. 2000; Zhang et al. 2021).

### Statistical and machine learning techniques-based analyses

Differences across the three diagnostic groups were studied with Pearson Chi-squared (in case of nominal independent variables) and Kruskal Wallis Chi-squared test (in case of continuous variables), since the assumption of normality was violated based on Shapiro–Wilk test. When a statistically significant difference was indicated based on Pearson’s chi-squared test, post hoc multiple comparisons were conducted with Fisher's exact tests with p-value adjusted based on Bonferroni correction. In case of statistically significant differences based on Kruskal Wallis Chi-squared test Dunn's post hoc tests are carried out on each pair of groups and the adjusted p-values were obtained.

Proportional odds logistic regression (POLR) models were employed in the entire study sample for studying the relationship between diagnostic groups (served as the ordinal dependent variable for the POLR models) and total GDS score, which is more commonly considered in clinical research and practice than GDS items separately, and MMSE, under consideration of age, sex, and education. For the sake of completeness, the POLR models considering GDS items separately were computed and their results are presented in the supplement. POLR models were employed, since MCI is an intermediate mental state between healthy cognitive ageing and dementia, particularly in clinical phenotypes caused by neurodegenerative disease such as AD (Albert et al. 2011; Vega and Newhouse 2014; Jongsiriyanyong and Limpawattana 2018; Alexopoulos et al. 2021b; Georgiou et al. 2023). Stratified repeated random subsampling (stratified bootstrap resampling) was used to recursive partitioning to training- and validation set (70/30 ratio) (James et al. 2005). The procedure was repeated 20,000 times and the results (parameters estimates over the training data sets and performance evaluation metrics over the training and the validation data sets) were then averaged over the splits. The machine learning technique, Adaptive Boosting classification algorithm (AdaBoost) was implemented in Python. AdaBoost uses the boosting technique as an Ensemble Method to improve the predictive power by converting several weak learners to strong learners (Zhu et al. 2009). It begins by fitting a classifier on the original dataset and then fits additional copies of the classifier on the same dataset but where the weights of incorrectly classified instances are adjusted such that subsequent classifiers focus more on cases in which they face difficulties in classifying them. The Synthetic Minority Oversampling Technique (SMOTE) was also applied to deal with the imbalanced datasets (Fernández et al. 2018). AdaBoost was employed after proper scaling, i.e. standardizing the quantitative variables and encoding the qualitative variables, for exploring the utility of GDS items in distinguishing between cognitively healthy individuals and patients with MCI or AD dementia (Lu et al. 2015). GDS items were separately considered, since machine learning techniques can discover complex relationships and patterns in data (Sarker 2021). Nonetheless, for the sake of completeness, AdaBoost models considering GDS total score were also computed, and their results are presented in the supplement. The classification performance of the regression- and AdaBoost models including the GDS was also compared to the accuracy of classifying study participants using the MMSE total score and the MMSE score-based rule of thumb (Fountoulakis et al. 2000; Zhang et al. 2021). The utility of a diagnostic test in clinical practice cannot be exclusively justified through high accuracy values, but the sensitivity and specificity values should be balanced (Van Stralen et al. 2009). Additionally, for highly imbalanced data sets, the F1 Score, which combines sensitivity and precision to one performance metric, is generally preferred from other metrics, such as the Area Under Curve (AUC), when there is a particular interest, as in this study, toward the classification of the minority class (see for example, (Gaudreault and Branco 2024)).

## Results

The study sample included *n* = 1737 participants without cognitive impairment, *n* = 334 with MCI, and *n* = 142 with AD dementia. Sociodemographic and clinical data are shown in Table 1. Age significantly differed across the diagnostic groups, with people with MCI or AD dementia being older compared to individuals without cognitive deficits. No significant differences in either education or sex distribution were detected. There were significant differences in GDS total scores between people with MCI or AD dementia and those without cognitive impairment, while the frequency of people with GDS scores indicating moderate to severe depression increased as cognitive impairment advanced (Table 1). People with MCI chose more often responses to twelve GDS items that point to the presence of depressive symptoms compared to participants without cognitive impairment and the difference between the groups was statistically significant. No differences were detected between these two groups in responses to the items related to worthlessness, loss of energy and suicidal thoughts. Statistically significant differences were observed between AD dementia and older individuals without cognitive deficits in responses to all GDS items except for the GDS item regarding “getting often bored” and the item pertaining to helplessness. Compared to people with MCI, older adults with AD dementia reported more frequently that they did not feel that it is wonderful to be alive and have feelings of hopelessness, worthlessness, memory difficulties. There was also a difference related to lack of energy between the two groups, but this difference did not reach statistical significance.

**Table 1:** Demographic and clinical data of the study sample

	Individuals without cognitive impairment (Group 1, G1)	Individuals with Mild Cognitive Impairment (Group 2, G2)	Individuals with Dementia (Group 3, G3)	Comparison of G1 = G2 = G3	Pairwise Comparisons		
				Value of test statistic (p-value)	G1–G2	G1–G3	G2–G3
N	1737	334	142				
Age, years*	73.28 (5.24) [65,91]	75.24 (5.49) [65,90]	79.08 (5.98) [65,100]	141.357^ǂǂ^ (< 0.001)	− 233.342 < 0.001^‡‡^	− 602.907 < 0.001^‡‡^	− 369.565 < 0.001^‡‡^
Sex (female, N, %)	1059 (61.0)	200 (59.9)	78 (54.9)	6.771^**ǂ**^ (0.034)	1^‡^	0.471^‡^	0.948^‡^
Education, (≤ 9 years, %)	1073 (61.8)	224 (67.1)	100 (70.4)	2.048^**ǂ**^ (0.359)	3.309 0.220^‡^	4.154 0.141^‡^	0.516 1^‡^
Mini Mental State Examination (MMSE)	0.235 (0.674) [− 4.642,0.954]	− 0.566 (0.946) [− 3.803, 0.954]	− 2.243 (1.499) [− 6.041, 0.674]	449.215^ǂǂ^ (< 0.001)	− 517.137 < 0.001^‡‡^	− 960.336 < 0.001^‡‡^	− 443.199 < 0.001^‡‡^
Geriatric depression scale (GDS) total score	1.76 (2.68) [0,12]	2.76 (3.22) [0,12]	3.31 (3.78) [0,12]	49.108^ǂǂ^ (< 0.001)	− 194.610 < 0.001^‡‡^	− 269.379 < 0.001^‡‡^	− 74.768 < 0.658^‡‡^
GDS total score indicating moderate to severe depression (GDS total score 9–15) (N, %)	60 (3.45)	21 (6.29)	20 (14.08)	36.959^**ǂ**^ (< 0.001)	6.360 0.006^‡^	36.443 < 0.001^‡^	7.368 0.003^‡^
GDS Item 1 (depression positive, %)	283 (16.3)	77 (23.05)	42 (29.6)	21.909^ǂ^ (< 0.001)	8.918 0.001^‡^	16.196 < 0.001^‡^	2.262 0.404^‡^
GDS Item 2 (depression positive, %)	419 (24.1)	112 (33.5)	62 (43.6)	34.652^ǂ^ (< 0.001)	13.013 < 0.001^‡^	26.315 < 0.001^‡^	4.408 0.114^‡^
GDS Item 3 (depression positive, %)	293 (16.86)	97 (29.04)	43 (30.28)	37.450^ǂ^ (< 0.001)	27.161 < 0.001^‡^	16.084 < 0.001^‡^	0.074 1^‡^
GDS Item 4 (depression positive, %)	346 (19.91)	87 (26.05)	37 (26.06)	8.396^ǂ^ (0.015)	6.363 0.045^‡^	3.046 0.251^‡^	< 0.001 1^‡^
GDS Item 5 (depression positive, %)	365(21.01)	113 (33.83)	54 (38.03)	41.469^ǂ^ (< 0.001)	25.930 < 0.001^‡^	21.934 < 0.001^‡^	0.770 1^‡^
GDS Item 6 (depression positive, %)	301 (17.32)	92 (27.54)	31 (21.83)	19.576^ǂ^ (< 0.001)	19.016 < 0.001^‡^	1.829 0.514^‡^	1.698 0.628^‡^
GDS Item 7 (depression positive, %)	332 (19.11)	114 (34.13)	47 (33.09)	46.754^ǂ^ (< 0.001)	37.392 < 0.001^‡^	15.945 < 0.001^‡^	0.048 1^‡^
GDS Item 8 (depression positive, %)	210 (12.09)	61 (18.26)	23 (16.19)	10.385^ǂ^ (0.006)	9.388 0.001^‡^	2.039 0.516^‡^	0.293 1^‡^
GDS Item 9 (depression positive, %)	398 (22.9)	110 (32.9)	48 (33.8)	21.029^ǂ^ (< 0.001)	15.196 < 0.001^‡^	8.599 0.016^‡^	0.034 1^‡^
GDS Item 10 (depression positive, %)	135 (7.77)	64 (19.16)	46 (32.39)	106.988^ǂ^ (< 0.001)	41.840 < 0.001^‡^	91.423 < 0.001^‡^	9.819 0.008^‡^
GDS Item 11 (depression positive, %)	92 (5.29)	28 (8.38)	24 (16.9)	**31.336**^**ǂ**^** (< 0.001)**	**4.890** **0.112**^**‡**^	30.520 < 0.001^‡^	7.430 0.028^‡^
GDS Item 12 (depression positive, %)	105 (6.05)	32 (9.58)	29 (20.42)	**41.561**^**ǂ**^** (< 0.001)**	**5.670** **0.066**^**‡**^	40.972 < 0.001^‡^	10.482 0.007^‡^
GDS Item 13 (depression positive, %)	304 (17.5)	73 (21.85)	47 (33.09)	22.465^ǂ^ (< 0.001)	3.568 0.189^‡^	21.022 < 0.001^‡^	6.679 0.033^‡^
GDS Item 14 (depression positive, %)	123 (7.08)	50 (14.97)	35 (24.65)	61.906^ǂ^ (< 0.001)	22.773 < 0.001^‡^	52.597 < 0.001^‡^	**6.362** **0.040**^**‡**^
GDS Item 15 (depression positive, %)	196 (11.28)	59 (17.66)	28 (19.71)	16.761^ǂ^ (< 0.001)	10.564 0.005^‡^	8.894 0.014^‡^	0.281 1^‡^

^*^Mean (standard deviation)[range]

^ǂǂ^Kruskal–Wallis test adjusted for ties

^ǂ^Pearson’s chi-square

^‡‡^Dunn’s post hoc test p-value after Kruskal–Wallis test

^**‡**^Pearson’s chi-square with Bonferroni adjusted p-value

Regression models, which were employed to investigate the relationship between cognitive diagnostic status and GDS total score and between cognitive diagnostic status and MMSE total score revealed that both GDS- and MMSE total scores were associated with the cognitive status of the participants (0.1322 [0.1092, 0.1547] and -0.4325 [-0.4653, -0.4920], respectively) (Table 2). The accuracy of the POLR models including the GDS total scores as independent variable (training sets: 0.7850 [0.7824, 0.7875], validations sets: 0.7842 [0.7771, 0.7892]) was inferior to that of the POLR models including the MMSE (training sets: 0.8177 [0.8096, 0.8263], validation sets: 0.8170 [0.7997,08342]). Nonetheless, the difference in accuracy between them did not exceed 5% either in the training- or in the validation datasets (Table 3). The overall performance of the POLR models was acceptable (accuracy close to 0.8). However, the performance evaluation metrics of the model including GDS total score were very low both in detecting people without cognitive impairment (specificity) (training sets: 0.022 [0.0090, 0.0420], validation sets: 0.0214 [0.0000, 0.0420]) and in identifying older adults with MCI (sensitivity) (training sets: 0.0055 [0.0000, 0.0171], validation sets: 0.0053 [0.0000, 0.0200]) or AD dementia (training sets: 0.0412 [0.0202, 0.0606], validation sets: 0.0403 [0.0000, 0.0930]) and clearly lower than the respective specificity- and sensitivity values of the model including MMSE total score as independent variable (Table 2). Of note, the utility of the regression model with GDS total scores as an independent variable, which is here presented, and of the model including responses to GDS items separately did not significantly differ (supplement, Table 1S).

**Table 2:** The averages of the parameters of the proportional odds logistic regression model along with their 95% bootstrap confidence intervals based on 20,000 stratified bootstrap training sets

		POLR with GDS as independent variable	POLR with MMSE as independent variable
95% bootstrap confidence intervals	Intercept 2|1*	− 9.3659 (− 10.4006, − 8.3737)	7.8642 (6.2006, 9.5992)
	Intercept 3|2*	− 10.8568 (− 11.9115, − 9.8472)	5.6179 (3.9800, 7.3188)
Covariates	Age	0.1059 (0.0935, 0.1188)	0.0398 (0.0238, 0.0558)
	Education	0.1128 (− 0.0332, 0.2631)	− 0.8582 (− 1.0493, − 0.6706)
	Sex	− 0.1355 (− 0.2872, 0.0140)	− 0.3033 (− 0.4785, − 0.1302)
	Instrument	0.1322 (0.1092, 0.1547)	− 0.4325 (− 0.4653, − 0.4029)

*Intercept *j*|(*j* − 1) denotes the intercept in $\log it(Y \ge j) = \log \left( {\frac{P(Y \ge j)}{{1 - P(Y \ge j)}}} \right)$ for Group *j* = 2,3, where *Y* represents the response variable of the patient’s diagnosis

GDS: 15-item Geriatric Depression Scale; MMSE: Mini Mental State Examination; POLR: Proportional odds logistic regression

**Table 3:** The performance evaluation metrics for the two proportional odds logistic regression models along with their 95% bootstrap confidence intervals based on 20,000 stratified bootstrap training and validation sets

	Training sets					Validation sets			
	Threshold	Accuracy	Sensitivity	Specificity	F1 score	Accuracy	Sensitivity	Specificity	F1 score
POLR with GDS as independent variable		0.7850 (0.7824, 0.7875)	G1: 0.9957 (0.9918, 0.9984)	G1: 0.0222 (0.0090, 0.0420)	G1: 0.8798 (0.8784, 0.8814)	0.7842 (0.7771, 0.7892)	G1: 0.9953 (0.9866, 1.0000)	G1: 0.0214 (0.0000, 0.0420)	G1: 0.8792 (0.8753, 0.8823)
			G2: 0.0055 (0.0000, 0.0171)	G2: 0.9961 (0.9909, 0.9992)	G2: 0.0145 (0.0082, 0.0328)		G2: 0.0053 (0.0000, 0.0200)	G2: 0.9961 (0.9893, 1.0000)	G2: 0.0247 (0.0187, 0.0561)
			G3: 0.0412 (0.0202, 0.0606)	G3: 0.9986 (0.9965, 1.0000)	G3: 0.0773 (0.0385, 0.1132)		G3: 0.0403 (0.0000, 0.0930)	G3: 0.9983 (0.9936, 1.0000)	G3: 0.0854 (0.0426, 0.1702)
POLR with MMSE as independent variable		0.8177 (0.8096, 0.8263)	G1: 0.9679 (0.9635, 0.9725)	G1: 0.3808 (0.3455, 0.4172)	G1: 0.9098 (0.9050, 0.9149)	0.8170 (0.7997, 0.8342)	G1: 0.9677 (0.9522, 0.9831)	G1: 0.3808 (0.3149, 0.4472)	G1: 0.9095 (0.8995, 0.9193)
			G2: 0.1768 (0.1408, 0.2140)	G2: 0.9494 (0.9433, 0.9559)	G2: 0.2412 (0.1974, 0.2848)		G2: 0.1744 (0.1099, 0.2444)	G2: 0.9490 (0.9323, 0.9651)	G2: 0.2372 (0.1565, 0.3172)
			G3: 0.3949 (0.3375, 0.4512)	G3: 0.9871 (0.9843, 0.9902)	G3: 0.4890 (0.4286, 0.5469)		G3: 0.3935 (0.2500, 0.5429)	G3: 0.9869 (0.9786, 0.9948)	G3: 0.4855 (0.3461, 0.6182)

GDS: 15-item Geriatric Depression Scale; G1: Cognitively healthy individuals; G2: Mild cognitive impairment; G3: Dementia due to Alzheimer’s disease LR: Logistic regression; MMSE: Mini Mental State Examination; POLR: Proportional odds logistic regression

The AdaBoost model yielded more balanced performance evaluation metrics compared to regression models (Table 4). Although the accuracy values of AdaBoost models were lower compared to the POLR models (AdaBoost model including GDS items: training sets 0.7292, validation sets: 0.7248; model including MMSE: training sets: 0.7259, validation sets: 0.6934) the sensitivity- and specificity values of both classifiers were more balanced resulting in higher F1 scores for the detection of MCI (model including GDS items: training sets: 0.6099, validation sets: 0.2935; model including MMSE: training sets: 0.5955, validation sets: 0.3754) and AD dementia (model including GDS items: training sets: 0.7661, validation sets: 0.2273; model including MMSE: training sets: 0.8278, validation sets: 0.5934) compared to the respective POLR models. The model with GDS items as independent variables had slightly higher average accuracy than the model with MMSE as independent variable, but specificity and sensitivity were more balanced in the latter model (Table 4). The metrics of AdaBoost models with GDS total scores as independent variable were slightly inferior to that of models considering responses to GDS items separately (supplement, Table 2S).

**Table 4:** The performance evaluation metrics for the Adaptive Boosting algorithm (AdaBoost), which uses the boosting technique as an Ensemble Method, in the training and validation sets

	Training sets				Validation sets			
	Accuracy	Sensitivity	Specificity	F1 score	Accuracy	Sensitivity	Specificity	F1 score
AdaBoost with Smote with GDS Items as independent variables	0.7292	G1: 0.8248	G1: 0.8548	G1: 0.7986	0.7248	G1: 0.8429	G1: 0.3189	G1: 0.8480
		G2: 0.5668	G2: 0.8495	G2: 0.6099		G2: 0.3140	G2: 0.8803	G2: 0.2935
		G3: 0.7937	G3: 0.8250	G3: 0.7661		G3: 0.2128	G3: 0.9241	G3: 0.2273
AdaBoost with Smote with MMSE as independent variable	0.7259	G1: 0.7224	G1: 0.8755	G1: 0.7471	0.6934	G1: 0.7223	G1: 0.7193	G1: 0.8086
		G2: 0.5971	G2: 0.7988	G2: 0.5955		G2: 0.5612	G2: 0.7338	G2: 0.3754
		G3: 0.8517	G3: 0.8591	G3: 0.8278		G3: 0.6585	G3: 0.9474	G3: 0.5934

GDS: 15-item Geriatric Depression Scale; G1: Cognitively healthy individuals; G2: Mild cognitive impairment; G3: Dementia due to Alzheimer’s disease; MMSE: Mini Mental State Examination

The classification accuracy of the regression- and AdaBoost models with MMSE or depressive symptoms as independent variables was higher than that of the rule of thumb commonly used for classifying the cognitive function of individuals based on their performance on the MMSE in clinical practice. More specifically, the accuracy of this rule of thumb was 58.41%. The sensitivity and specificity in detecting MCI was 44.70% and 67.44%, respectively and in identifying AD dementia was 84.07% and 88.58%, respectively.

## Discussion

The present study sheds light on the potential utility of commonly detected depressive symptoms in contributing to the identification of MCI and AD dementia in older adults. The study and its findings expand previous literature as they (i) show that despite imbalances in performance evaluation metrics, the GDS tends to have a classification accuracy comparable to that of the widely used cognitive screening test MMSE particularly when machine learning techniques are employed, (ii) apply the AdaBoost machine learning technique, and (iii) consider each depression scale item separately in addition to the conventional strategy of considering depression scale total scores.

The findings yielded by the AdaBoost models showed that GDS items can differentiate between cognitively healthy ageing and both MCI and AD dementia with an accuracy similar to that of MMSE total score and superior to the MMSE score-based rule of thumb commonly used in clinical practice. Considering that the MMSE is one of the most widely used cognitive screening tests among older people, these results point to the potential utility of the GDS for identifying older individuals with possible cognitive impairment. Compared to the MMSE, which needs to be administered by trained staff, the GDS can be administered within approximately five minutes, even in the waiting room of a medical facility with minimal support of nonmedical staff (Costa et al. 2016); as such it could be easily used as a quick and rough screening tool in GP practices and primary healthcare centres. Provided the observations of our study are replicated in independent cohorts and balanced evaluation metrics are found, classification tools relying on machine learning techniques and GDS items may become in the future a low-cost, time efficient approach to identify cognitive impairment in ageing in community settings.

Compared to the regression models, the boosting technique using an Ensemble Method showed in most cases more balanced classification performance, as indicated by F1- values (Ndichu et al. 2023), albeit with lower accuracy values. F1 is a better metric to assess the quality of classification models when the classes are imbalanced and there is a serious downside to predicting false negatives values, since a model tends to be biased towards majority class samples (Ndichu et al. 2023). The disproportionate classification values in POLR models may also be partially attributed to the diagnostic uncertainties related to the distinguishment between MCI and dementia in clinical practice (Knopman and Petersen 2014; Lee 2023). The more balanced classification performance of AdaBoost models may be attributed to the fact that traditional classification models like regression models tend to ignore the imbalanced data issue, leading to poor classification performance in imbalanced datasets (Shahri et al. 2021). Moreover, AdaBoost models can make predictions through discovering generalizable nonlinear latent patterns between variables.

Depressive symptoms significantly differed between the diagnostic groups with higher GDS total scores among individuals with cognitive impairment. These observations are in line with consistent evidence pointing to higher frequency of depressive symptoms in people with MCI and AD dementia (Ismail et al. 2017; Leung et al. 2021). Although the statistically significant differences in responses to GDS items between individuals without cognitive impairment and older adults with MCI or AD dementia were related to affective (e.g. pessimism, loss of interest), cognitive (e.g. worthlessness, dislike of self), and somatic symptoms (e.g. loss of energy, concentration difficulties) (O’Shea et al. 2018), the symptoms that differed between MCI and AD dementia were mainly cognitive and somatic and not affective. This observation is in accordance with previous studies pointing out that progression of cognitive impairment is accompanied by physical activity decline and loss of self-esteem (Hu et al. 2016; Scott 2022), while sadness may not be the most prominent depressive symptom in people with dementia (Helvik et al. 2019). The observed complex patterns of variation of responses to the different scale items across the study groups underscore the necessity of employing analytical methods able to discover complex relationships between items and devoted to maximizing classification performance. Furthermore, they illustrate that the exclusive focus on depression severity may deprive the input of individual depressive symptoms in distinguishing between healthy cognitive ageing and cognitive impairment of valuable information.

Depressive symptoms and other neuropsychiatric symptoms (e.g. anxiety, apathy, delusions, and hallucinations) are a group of symptoms of the phenotype of both MCI and dementia. They affect up to 90% of people with dementia over the course of the syndrome, while they are present in 35–85% of individuals with MCI (Martin and Velayudhan 2020; Laganà et al. 2022; Saari 2022). Interestingly, neuropsychiatric symptoms have been shown to be related to AD biomarkers (Ng et al. 2021; Spampinato et al. 2023) and the total score of the Neuropsychiatric Inventory Questionnaire (NPI-Q) has been recently found to pertain to staging of cognitive impairment in ageing ((González et al. 2024)). Nonetheless, their potential contribution to detecting and diagnosing MCI and dementia has not been thoroughly studied yet (And et al. 2022). Despite the detected imbalances in performance evaluation metrics, our observations yield first evidence of the potential utility of depressive symptoms in the diagnostic workup of neurocognitive disorders.

This study has several limitations. First, depressive symptoms were captured with the GDS, a valid and reliable instrument, which, however, may have led to an overestimation of the presence of depressive symptoms (Thombs et al. 2018; Shin et al. 2019). Another limitation is that although the frequency of cognitive impairment was in line with previous reports (Nichols et al. 2022), the study sample was imbalanced with regard to people with MCI or AD dementia and those without cognitive impairment. To address this imbalance, the cohort was expanded beyond participants of HELIAD. Additional individuals evaluated at two old age psychiatry university outpatient clinics were included to enrich the sample. Furthermore, psychotropic medication (e.g. antidepressants, cholinesterase inhibitors) was not considered in the analyses, since GDS items are treated here as a pragmatic, simple tool to triage trajectories of cognitive decline in ageing screening tool that can be administered with minimal support of nonmedical staff. The diagnostic workup of the study sample is based on an extensive diagnostic workup which is not compatible with community-based healthcare. Thus, the utility of GDS items as an easy and quick screening tool warrants investigation in community settings, since the administration of the GDS precisely in such settings could be of great value in giving guidance to general practitioners for the necessity of further diagnostic steps. Finally, the clinical diagnoses of dementia and MCI were based on international diagnostic criteria. Nevertheless, the clinical diagnoses are neither always confirmed at autopsy nor always supported by biomarker constellations typical for AD (Georgiou et al. 2023). Thus, possibly erroneous clinical assessments should be also taken into account.

The findings of the study provide initial evidence that depressive symptoms may contribute to distinguishing between cognitive impairment and cognitively healthy ageing. Since depressive symptoms can be easily captured through brief depression scales in primary care clinical settings, they may contribute to directing older patients with cognitive complaints to targeted diagnostic pathways, especially in the dawn of disease-modifying treatments and in the absence of established non-invasive and affordable biomarkers. Nonetheless, the detected imbalances in performance evaluation metrics point out that the clinical usefulness of GDS items in detecting cognitive impairment should be treated with caution. Before final conclusions can be drawn, further studies using behavioural instruments other than the GDS, considering non- AD dementias and/or including AD biomarkers are needed in order to shed light on the potential clinical utility of such findings.

## Supplementary Information

Below is the link to the electronic supplementary material.

Supplementary file1 (DOCX 18 KB)

## Data Availability

The dataset used and analysed during the current study is available from the corresponding author on reasonable request.
