# Supplementary material for: The potential of depressive symptoms to identify cognitive impairment in ageing
Source: Eur J Ageing. 2025 Feb 25;22(1):7. doi: 10.1007/s10433-025-00837-1 (PMC11861444; doi:10.1007/s10433-025-00837-1)
Supplement: Supplementary file 1 — Supplementary file1 (DOCX 18 KB) [file 10433_2025_837_MOESM1_ESM.docx]

**The potential of depressive symptoms** **to** **identify cognitive impairment in aging**

Panagiotis Alexopoulos, Christos Bountoulis, Everina Katirtzoglou, Mary H Kosmidis, Kostas Siarkos, Mary Yannakoulia, Efthimios Dardiotis, Maria Skondra, Georgios Hadjigeorgiou, Robert Perneczky, Paraskevi Sakka, Eleni-Zacharoula Georgiou, Μarina Charalampopoulou, Panagiotis Felemegkas, Iracema Leroi, Apostolos Batsidis, Laura Perna, Antonios Politis, Nikolaos Scarmeas, Polychronis Economou

Table 1S. The performance evaluation metrics for the proportional odds logistic regression models (with the 15 items of the Geriatric depression scale considered as independent variables along with their 95% bootstrap confidence intervals based on 20,000 stratified bootstrap training and validation sets. 95% bootstrap confidence intervals based on 20,000 stratified bootstrap training and validation sets.

|  | Training sets | | | | | Validation sets | | | |
| --- | --- | --- | --- | --- | --- | --- | --- | --- | --- |
|  | Threshold | Accuracy | Sensitivity | Specificity | F1 score | Accuracy | Sensitivity | Specificity | F1 score |
| POLR  with GDS Items as independent variables |  | 0.7871 (0.7829, 0.7915) | G1: 0.9892 (0.9844, 0.9942) | G1: 0.0756 (0.0450, 0.1111) | G1: 0.8822 (0.8794, 0.8854) | 0.7840 (0.7738, 0.7937) | G1: 0.9868 (0.9731, 0.9962) | G1: 0.0682 (0.0350, 0.1049) | G1: 0.8800 (0.8740, 0.8853) |
|  |  |  | G2: 0.0268 (0.0085, 0.0470) | G2: 0.9871 (0.9802, 0.9932) | G2: 0.0486 (0.0160, 0.0840) |  | G2: 0.0248 (0.0000, 0.0600) | G2: 0.9872 (0.9769, 0.9964) | G2: 0.0484 (0.0180, 0.1053) |
|  |  |  | G3: 0.1034 (0.0606, 0.1414) | G3: 0.9967 (0.9938, 0.9993) | G3: 0.1788 (0.1111, 0.2393) |  | G3: 0.0940 (0.0233, 0.1628) | G3: 0.9954 (0.9871, 1.0000) | G3: 0.1612 (0.0435, 0.2759) |
| GDS: 15-item Geriatric Depression Scale; G1: Cognitively healthy individuals; G2: Mild cognitive impairment; G3: Dementia due to Alzheimer’s disease; MMSE: Mini Mental State Examination; POLR: Proportional odds logistic regression. | | | | | | | | | |

Table 2S The performance evaluation metrics for the Adaptive Boosting algorithm (AdaBoost) models including total scores on the 15-item Geriatric depression scale as independent variable.

|  | Training sets | | | | Validation sets | | | |
| --- | --- | --- | --- | --- | --- | --- | --- | --- |
|  | Accuracy | Sensitivity | Specificity | F1 score | Accuracy | Sensitivity | Specificity | F1 score |
| AdaBoost with Smote with GDS total score as independent variable | 0.6884 | G1: 0.7466 | G1: 0.8525 | G1: 0.7517 | 0.6525 | G1: 0.7465 | G1: 0.4272 | G1: 0.8004 |
|  |  | G2: 0.5637 | G2: 0.8028 | G2: 0.5805 |  | G2: 0.2551 | G2: 0.7975 | G2: 0.2252 |
|  |  | G3: 0.7513 | G3: 0.7939 | G3: 0.7271 |  | G3: 0.4634 | G3: 0.8627 | G3: 0.3089 |
| GDS: 15-item Geriatric depressions scale; G1: Cognitively healthy individuals; G2: Mild cognitive impairment; G3: Dementia due to Alzheimer’s disease ΄ | | | | | | | | |
